# Distribution and Phylogeny of EFL and EF-1α in Euglenozoa Suggest Ancestral Co-Occurrence Followed by Differential Loss

**DOI:** 10.1371/journal.pone.0005162

**Published:** 2009-04-09

**Authors:** Gillian H. Gile, Drahomíra Faktorová, Christina A. Castlejohn, Gertraud Burger, B. Franz Lang, Mark A. Farmer, Julius Lukeš, Patrick J. Keeling

**Affiliations:** 1 Canadian Institute for Advanced Research and Department of Botany, University of British Columbia, Vancouver, British Columbia, Canada; 2 Biology Centre, Institute of Parasitology, Czech Academy of Sciences and Faculty of Natural Sciences, University of South Bohemia, České Budějovice, Czech Republic; 3 Department of Cellular Biology, The University of Georgia, Athens, Georgia, United States of America; 4 Centre Robert Cedergren, Bioinformatics & Genomics, Département de biochimie, Université de Montréal, Montréal, Quebec, Canada; Yale University, United States of America

## Abstract

**Background:**

The eukaryotic elongation factor EF-1α (also known as EF1A) catalyzes aminoacyl-tRNA binding by the ribosome during translation. Homologs of this essential protein occur in all domains of life, and it was previously thought to be ubiquitous in eukaryotes. Recently, however, a number of eukaryotes were found to lack EF-1α and instead encode a related protein called EFL (for EF-Like). EFL-encoding organisms are scattered widely across the tree of eukaryotes, and all have close relatives that encode EF-1α. This intriguingly complex distribution has been attributed to multiple lateral transfers because EFL's near mutual exclusivity with EF-1α makes an extended period of co-occurrence seem unlikely. However, differential loss may play a role in EFL evolution, and this possibility has been less widely discussed.

**Methodology/Principal Findings:**

We have undertaken an EST- and PCR-based survey to determine the distribution of these two proteins in a previously under-sampled group, the Euglenozoa. EF-1α was found to be widespread and monophyletic, suggesting it is ancestral in this group. EFL was found in some species belonging to each of the three euglenozoan lineages, diplonemids, kinetoplastids, and euglenids.

**Conclusions/Significance:**

Interestingly, the kinetoplastid EFL sequences are specifically related despite the fact that the lineages in which they are found are not sisters to one another, suggesting that EFL and EF-1α co-occurred in an early ancestor of kinetoplastids. This represents the strongest phylogenetic evidence to date that differential loss has contributed to the complex distribution of EFL and EF-1α.

## Introduction

The essential eukaryotic translation elongation factor EF-1α and its distantly related paralog EFL (for EF-Like) are GTPases with a complex, mutually exclusive distribution. While EF-1α is well known from plants, animals, and fungi, and has been characterized at the structural [1] and functional [2] levels, EFL was discovered more recently in a small number of single-celled eukaryotes that were found to lack EF-1α [3]. EFL is considered likely to perform the same canonical translation function as EF-1α due to their mutually exclusive distribution and the observation that EF-1α's binding sites for EF-1β, aminoacyl-tRNAs, and GTP are conserved in EFL [3], though no functional analyses of EFL have been carried out. Curiously, EFL-encoding lineages are scattered across the tree of eukaryotes, such that they are each more closely related to an EF-1α-encoding lineage than they are to one other. This complex pattern has persisted despite further studies of EFL in green algae [4], fungi [5], ichthyosporids [6], [7], cryptophytes, haptophytes, red algae [8], [9], and diatoms [10] that have greatly expanded its known distribution. In general, the phylogeny of EFL is incongruent with the phylogeny of the organisms in which it is found, which is not consistent with a single ancestral origin of eukaryotic EFL genes. As a result, multiple lateral gene transfers are often invoked to explain the complex distribution of EFL, despite the lack of compelling evidence for this interpretation. Only in one case did the phylogeny of EFL reveal a potential donor lineage for the putative lateral gene transfer [10]. In addition to lateral gene transfer, differential loss of EFL and EF-1α is a mechanism that can explain the unusual distribution of these two proteins. This possibility has not been explored as fully, although a close examination of the distribution of EFL in green algae pointed to this as a contributing factor in that lineage [4].

A clearer picture of the evolutionary history of EFL and EF-1α will depend on greater sampling, both on a broad scale to determine their distribution in eukaryotes as a whole and on a finer taxonomic scale in lineages where both proteins are found to gain insight into the processes behind this distribution. As part of an ongoing effort to address both these levels of sampling, we have undertaken an EST- and PCR-based survey to determine the distribution of EFL and EF-1α in a previously under-sampled group, the Euglenozoa. The Euglenozoa are a phylum of protists with diverse habitats and lifestyles belonging to the somewhat contentious supergroup Excavata [11], [12] and comprised of three major lineages: Euglenida, Kinetoplastea, and Diplonemida. There are approximately 1000 described species of euglenids, including the well-known *Euglena gracilis*, a photoautotrophic freshwater protist, and other non-photosynthetic bacteriovores, eukaryovores, and osmotrophs [13]. Kinetoplastids, which include human parasites of the genera *Trypanosoma* and *Leishmania*, are characterized by the complex masses of DNA, known as kinetoplasts, found in their mitochondria [14]. There are only two described genera of diplonemids, although deep-sea environmental studies of small subunit ribosomal RNA (SSU rRNA) sequences have revealed considerable genetic diversity and two novel clades within this group [15]. Within the Euglenozoa, the kinetoplastids and diplonemids are considered most likely to be sisters to the exclusion of euglenids 16, 17, although they are separated by a great evolutionary distance [18].

Prior to this study, EF-1α sequences were known only from *E. gracilis* and a few of the medically important *Trypanosoma* and *Leishmania* species, and EFL was not known from any member of the Euglenozoa or even the excavate supergroup to which they belong. In the present study, we have examined 24 species spanning the phylogenetic diversity of Euglenozoa for the presence of EFL and EF-1α. EFL was found in 6 species scattered among all three euglenozoan lineages, whereas EF-1α was found in the remaining 18 species, but not from any diplonemid. None of the species examined was found to encode both proteins. The monophyly of euglenozoan EF-1α and close evolutionary similarity between EFL from *Neobodo saliens* and *Trypanoplasma borreli*, two kinetoplastids from distinct clades [16], [19], [20] suggest that, at least in the kinetoplastids, this pattern is due to differential loss from an ancestral state of co-occurrence. Although we cannot rule out the unlikely possibility that lateral gene transfer produced this pattern, this is the clearest phylogenetic evidence from any group to date that differential loss has contributed to the complex distribution of EFL and EF-1α.

## Materials and Methods

### Culture sources and nucleic acids extraction

Three diplonemid species, five euglenid species, and sixteen kinetoplastid species were tested for the presence of EFL and EF-1α by PCR, RT-PCR, or by searching EST libraries. Cell isolation and nucleic acids extraction methods were described previously for the diplonemids *Diplonema ambulator* ATCC 50223 and *Diplonema papillatum* ATCC 50162 [21], and *Rhynchopus euleiides* ATCC 50226 [22], [23], the euglenids *Entosiphon sulcatum* [24], *Peranema trichophorum* CCAP 1260/1 B and *Petalomonas cantuscygni* CCAP 1259/1 [23], and the kinetoplastids *Blastocrithidia culicis* ATCC 30268, *Herpetomonas muscarum* ATCC 30260, *Herpetomonas pessoai* ATCC 30252 [25], *Leishmania tarentolae* strain UC [26], *Leptomonas bifurcata* [27], *Leptomonas costaricensis* [28], *Leptomonas podlipaevi* [29], *Neobodo saliens* (syn. *Bodo saliens*) ATCC 50358 [30], *Perkinsiella amoebae,* along with its host *Neoparamoeba branchiphila* strain AMOP1 [31], *Trypanoplasma borreli* strain Tt-JH [32], *Trypanosoma avium* [33], and *Trypanosoma brucei equiperdum* strain STIB818 [34]. The remaining four species were ordered from culture collections: *Rhynchobodo sp.* ATCC 50359, *Dimastigella trypaniformis* ATCC 50263, *Bodo saltans* CCAP 1907/2, and *Rhynchomonas nasuta* strain AZ-4 ATCC 50292. Total RNA was extracted from *Rhynchomonas nasuta* using the RNeasy Plant Mini Kit (Qiagen), and from *Trypanoplasma borreli* using Trizol reagent (Invitrogen). Genomic DNA was extracted from *Rhynchobodo sp*., *B. saltans*, and *D. trypaniformis* using the DNeasy Plant Mini Kit (Qiagen).

### EST identification and assembly

EST libraries were generated as described [35]. EFL sequences from *D. ambulator*, *D. papillatum*, and *R. euleiides* and EF-1α sequences from three euglenids, *Astasia longa*, *Euglena gracilis,* and *P. trichophorum*, and seven non-euglenozoan excavates, *Histiona aroides*, *Jakoba bahamiensis*, *Jakoba libera*, *Malawimonas californiana*, *Reclinomonas americana*, *Seculamonas ecuadoriensis*, and *Stachyamoeba lipophora* were identified by tBLASTn search in the taxonomically broad EST database (TBestDB, http://amoebidia.bcm.umontreal.ca/pepdb/searches/login.php). Contigs of several ESTs were assembled using Sequencher 4.5 (GeneCodes) and examined for quality before export and conceptual translation of consensus sequences.

### Primer sets and sequencing

All non-EST sequences generated in this study were amplified from genomic DNA except for *R. nasuta*, which was amplified from cDNA. EF-1α sequences were amplified using nested degenerate primer pairs EF1a F1 and EF1a R1 followed by EF+ F2 and EF1a R2, except for sequences from *B. culicis*, *H. muscarum*, and *T. brucei equiperdum* which were amplified using EF1a F1 and EF1a Rc, and *B. saltans*, *D. trypaniformis*, *Rhynchobodo sp*., and *R. nasuta*, which were amplified using the degenerate primers EUG EF1a 1F and EUG EF1a 1R or 2R (Table 1). EFL from *N. saliens* was amplified using nested degenerate primer pairs EFL F1 and EFL R1 followed by EF+ F2 and EFL R2. EFL from *T. borreli* was amplified from genomic DNA with primers EFL F1 and EFL Rc, and subsequently confirmed by RT-PCR from total RNA using primers specific to the spliced leader RNA sequence and EFL sequence (data not shown). All templates were tested for both EFL and EF-1α, and none were found to encode both proteins. PCR products from *E. sulcatum, H. pessoai, L. tarentolae, P. amoebae, P. cantuscygni, R. nasuta*, and *T. avium* were TOPO-TA cloned into pCR 2.1 vector (Invitrogen) and sequenced on both strands. All other PCR products were sequenced directly on both strands. New sequences obtained in this study (Table 2) were deposited in GenBank under accession numbers FJ807237-FJ807268.

**Table 1:** Names and sequences of primers used in this study.

Name	Sequence, 5′ to 3′
EFL F1	CTGTCGATCGTCATHTGYGGICAYGTHGA
EFL R1	GAACGCGATTCGGGATARNCCYTCRCA
EF+ F2	CATGTCGATGCAGGTAAGTCNACNACNACNGG
EFL R2	CTTCTTTCCTCCAGTYTCYTTNCC
EFL Rc	CTTGATRTTIAGICCIACRTTRTCNCC
EF1a F1	AACATCGTCGTGATHGGNCAYGTNGA
EF1a R1	ACGCCAACTGCTACNGTYTGNCKCAT
EF1a R2	CTGTCCAGGATGGTTCATDATDATNACYTG
EF1a Rc	CTTGATCACICCIACIGCNACNGT
EUG EF1a 1F	GGGIAARGAIAARGTICAYATNARYYT
EUG EF1a 1R	NCCNARIGGIGSRTARTCIKTRAA
EUG EF1a 2R	CCNACNGCIACITGYYGICGCATRTC

**Table 2:** New sequences obtained in this study.

Species	EFL/EF-1α	Method
Diplonemids		
*Diplonema ambulator* ATCC 50223	EFL	ESTs
*Diplonema papillatum* ATCC 50162	EFL	ESTs
*Rhynchopus euleiides* ATCC 50226	EFL	ESTs
Kinetoplastids		
*Blastocrithidia culicis* ATCC 30268	EF-1α	PCR
*Bodo saltans* CCAP 1907/2	EF-1α	PCR
*Dimastigella trypaniformis* ATCC 50263	EF-1α	PCR
*Herpetomonas muscarum* ATCC 30260	EF-1α	PCR
*Herpetomonas pessoai* ATCC 30252	EF-1α	PCR
*Leishmania tarentolae* UC strain	EF-1α	PCR
*Leptomonas bifurcata*	EF-1α	PCR
*Leptomonas costaricensis*	EF-1α	PCR
*Leptomonas podlipaevi*	EF-1α	PCR
*Neobodo saliens* ATCC 50358	EFL	PCR
*Perkinsiella amoebae*	EF-1α	PCR
*Rhynchobodo sp.* ATCC 50359	EF-1α	PCR
*Rhynchomonas nasuta* strain AZ-4 ATCC 50292	EF-1α	RT-PCR
*Trypanoplasma borreli* strain Tt-JH	EFL	PCR
*Trypanosoma avium*	EF-1α	PCR
*Trypanosoma brucei equiperdum* strain STIB818	EF-1α	PCR
Euglenids		
*Astasia longa*	EF-1α	ESTs
*Entosiphon sulcatum*	EF-1α	PCR
*Euglena gracilis*	EF-1α	ESTs
*Peranema trichophorum* CCAP 1260/1 B	EF-1α	ESTs
*Petalomonas cantuscygni* CCAP 1259/1	EFL	PCR
Heterolobosean		
*Stachyamoeba lipophora*	EF-1α	ESTs
Jakobids		
*Histiona aroides*	EF-1α	ESTs
*Jakoba bahamiensis*	EF-1α	ESTs
*Jakoba libera*	EF-1α	ESTs
*Reclinomonas americana*	EF-1α	ESTs
*Seculamonas ecuadoriensis*	EF-1α	ESTs
*Malawimonas*		
*Malawimonas californiana*	EF-1α	ESTs
Amoebozoan		
*Neoparamoeba sp.*	EF-1α	PCR

### Phylogenetic analysis

New and previously published EFL and EF-1α sequences were translated and aligned using MAFFT [36] and edited in MacClade 4.08 [37] to final matrix sizes of 43 taxa and 478 characters for EFL and 51 taxa and 428 characters for EF-1α. In addition to these datasets, the EF-1α phylogeny was inferred with the anomalous, long-branch sequence from the heterolobosean *Acrasis rosea* (GenBank accession AAG48934) included. EFL phylogenies were also inferred from an alignment with the 7 longest branches excluded: *Ditylum brightwellii, Thalassiosira pseudonana, Reticulomyxa filosa, Planoglabratella opercularis, Goniomonas amphinema,* and cytosolic sequences from *Bigelowiella natans* and *Gymnochlora stellata* (data not shown).

Phylogenetic trees were inferred using maximum likelihood (ML) and Bayesian methods. ProtTest 1.4 [38] ranked RtREV the best amino acids substitution model for both proteins. ML trees were inferred with RAxML 7.0.4 [39] and PhyML 3.0 [40] using RtREV and LG amino acids substitution matrices, respectively [41], [42], and using four rate categories approximated by a Γ distribution, with parameter α, amino acids frequencies, and proportion of invariable sites estimated from the data. Five hundred bootstrap replicates were performed in each program for each dataset. PhyloBayes 2.3 [43] was used to perform Bayesian analyses using the CAT model [44] with 4 discrete Γ categories. For each dataset, two independent chains were run for 112,000 cycles, saving one tree in ten. The first 200 trees (representing 2000 cycles) were discarded as burn-in, and the remaining 11,000 trees from each chain in each dataset were used to test for convergence and compute the 50% majority rule consensus tree. Maxdiff values were 0.044 and 0.072 for EFL with long branches included and excluded, respectively, and 0.044 and 0.054 for EF-1α including and excluding the *A. rosea* sequence.

Approximately Unbiased (AU) tests [45] were carried out to evaluate the likelihood of alternate EFL topologies in which euglenozoan sequences are constrained as monophyletic. Site-likelihoods for these trees were calculated by RAxML [39] using the RtREV amino acids substitution model [41] and four Γ rate categories with parameter α, amino acid frequencies, and the proportion of invariable sites estimated from the data. AU tests were performed using CONSEL 1.19 [46].

## Results

### Distribution of EFL and EF-1α

Previously, only EF-1α sequences were known in the Euglenozoa from *Trypanosoma* and *Leishmania* species and *E. gracilis*. We examined 24 species spanning the phylogenetic diversity of the Euglenozoa as well as 7 non-euglenozoan excavate species for the presence of EFL and EF-1α by PCR or by searching EST libraries (Table 2). EFL was found in the diplonemids *D. ambulator*, *D. papillatum*, and *R. euleiides*, two deep-branching kinetoplastids *N. saliens* and *T. borreli*, and *P. cantuscygni*, a deep-branching euglenid [24]. All other species were found to encode EF-1α, including *N. branchiphila*, the amoebozoan host of *P. amoebae*, with which its DNA was co-purified. None of the species examined were found to encode both proteins, although this possibility cannot be ruled out. Where complete euglenozoan genomes exist, for the kinetoplastids *Trypanosoma brucei, Trypanosoma cruzi*, *Leishmania braziliensis*, *Leishmania infantum*, and *Leishmania major* [47]–[50] we can confirm that they each encode only EF-1α. To date there are only two documented cases of EFL and EF-1α co-occurrence: both genes were amplified by PCR in the zygomycete fungus *Basidiobolus ranarum* [5], and both are found in the complete genome of the diatom *Thalassiosira pseudonana*. While no expression data is available for the former, in the latter only EFL is expressed [10].

### Phylogenetic analyses of EF-1α and EFL

**Figure 1:**
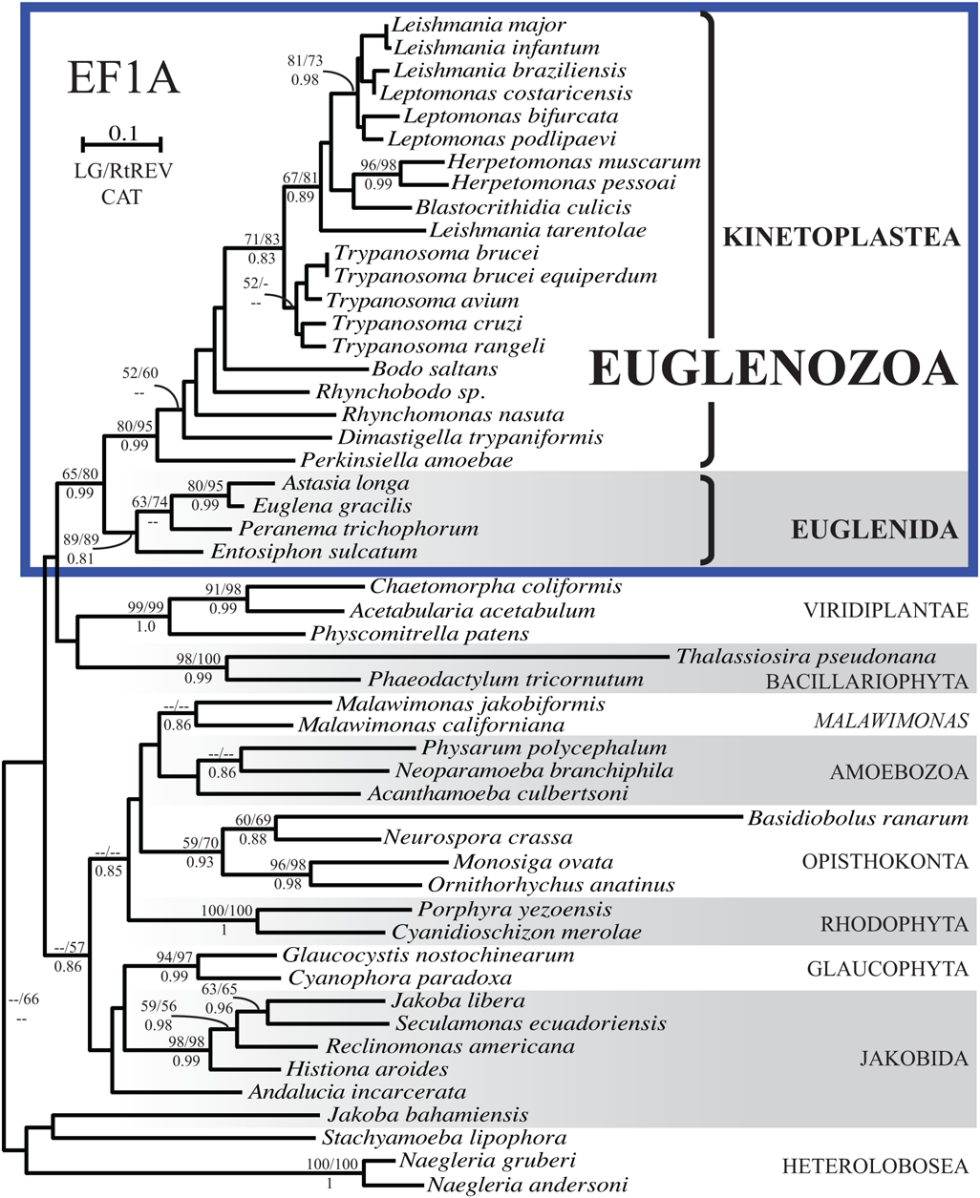
Maximum likelihood phylogeny of EF-1α including Bayesian posterior probabilities. The tree was inferred under LG, RtREV, and CAT amino acids substitution models using 4 Γ categories plus invariable sites; the LG topology is displayed. Bootstrap support greater than 50% and Bayesian posterior probabilities greater than 0.8 are displayed at nodes, with LG/RtREV ML bootstrap values above and CAT model posterior probability below. Euglenozoan taxa are boxed in blue.

The phylogeny of EF-1α is broadly concordant with accepted euglenozoan relationships. The monophyly of kinetoplastids, euglenids, and Euglenozoa as a whole are recovered with moderate to good support depending on the method (Fig. 1). Within the euglenids, the branching order of genera was also consistent among methods and consistent with current hypotheses for the organismal phylogeny. The branching order within the kinetoplastids in ML trees roughly matches expectations but without support, and with the major exception that *R. nasuta* and *D. trypaniformis* did not form a clade, although they consistently group together in other published analyses [20], [51]–[54]. The overall prevalence of EF-1α in the Euglenozoa and its broad congruence with accepted organismal relationships suggest that EF-1α was present in the common ancestor of this group.

Preliminary EF-1α analyses were carried out with the EF-1α sequence from the heterolobosean *Acrasis rosea* (GenBank accession AAG48934) included. The position of this sequence was not resolved: rather than branching with other heteroloboseans, it formed a long branch within the *Herpetomonas* clade in ML analyses and at the base of kinetoplastids in the Bayesian analysis, and its inclusion reduced bootstrap support for trypanosomatid, kinetoplastid, and euglenozoan monophyly. Because of its uncertain placement, its disruptive effect on resolution throughout the kinetoplastid clade, and the fact that *A. rosea* is not a euglenozoan, this sequence was removed from the alignment for further analysis.

**Figure 2:**
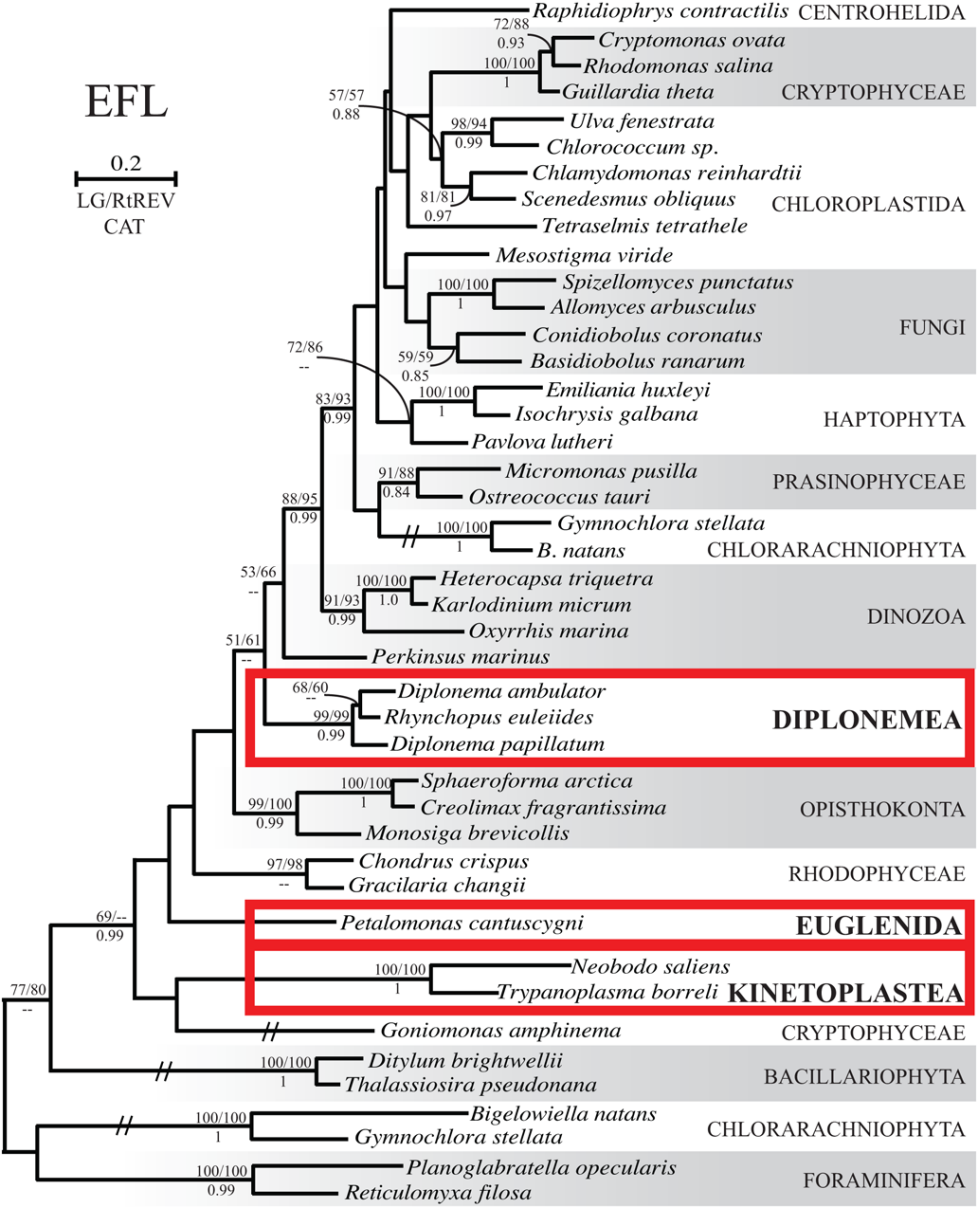
Maximum likelihood phylogeny of EFL including Bayesian posterior probabilities. The tree was inferred under LG, RtREV, and CAT amino acids substitution models using 4 Γ categories plus invariable sites; the LG topology is displayed. Bootstrap support greater than 50% and Bayesian posterior probabilities greater than 0.8 are displayed at nodes, with LG/RtREV ML bootstrap values above and CAT model posterior probability below. Branches with hatch marks are displayed at one half their actual length. Euglenozoan taxa are boxed in red.

**Figure 3:**
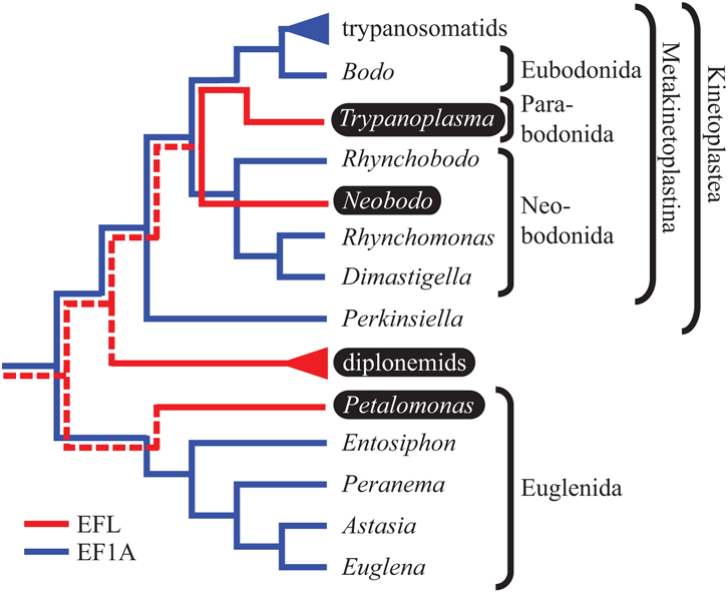
Schematic tree illustrating currently accepted phylogenetic relationships among euglenozoan taxa examined in this study. The presence of EFL (red) and EF-1α (blue) are traced along the organismal phylogeny to their origins with solid lines where there is phylogenetic evidence for their monophyly. Dotted lines hypothetically trace the presence of EFL back to the ancestor of Euglenozoa. Taxa shown in white text on black background encode EFL; all others encode EF-1α.

EFL phylogenies were inferred using the same models used for EF-1α. While much of the tree remains unresolved in all analyses, as is typical of EFL trees [3], [4], [8]–[10], three features emerge that are pertinent to the origin and evolution of EFL in the Euglenozoa (Fig. 2). First, the three lineages of euglenozoan EFL, diplonemids, kinetoplastids, and *P. cantuscygni*, never branch together. However, their positions are not clearly resolved, none of the nodes that separate them are supported, and the relative branching order of the three euglenozoan EFL lineages, *Goniomonas amphinema*, *Perkinsus marinus*, red algae, and a group of opisthokonts, varies greatly depending on the dataset analyzed and evolutionary model employed. Second, diplonemid EFL sequences robustly branch together in all analyses, suggesting that EFL is ancestral in this group. Third, and most importantly, the two kinetoplastid EFL sequences branch together with complete support in all analyses, providing strong evidence that EFL was present in their common ancestor as well. This is significant because *N. saliens* and *T. borreli* are members of two different subgroups in organismal phylogenies of kinetoplastids [16], [20], [51], [52], [55], which therefore places EFL at least as far back as the common ancestor of all kinetoplastids save the earliest-branching lineage that includes *P. amoebae* (Fig. 3). Because the phylogeny of EF-1α suggests that this protein was also present in the ancestor of kinetoplastids, we infer that both genes must have co-existed through much of early kinetoplastid evolution, and it therefore appears that the complex distribution of EFL and EF-1α in the kinetoplastids is likely due to differential loss.

To test the possibility that EFL sequences from the three euglenozoan lineages are monophyletic, we carried out approximately unbiased (AU) tests to evaluate alternative topologies in which their monophyly was constrained. For each of four ML topologies, a monophyletic euglenozoan clade in which kinetoplastids and diplonemids are sisters was grafted onto the positions where each of the three euglenozoan EFL lineages had individually branched in ML analyses. In tests including the entire dataset, euglenozoan EFL monophyly is not rejected at the 5% level when grafted to the diplonemid branch, but all other alternate topologies are rejected. Because significant rate heterogeneity is known in several EFL lineages, we also tested euglenozoan EFL monophyly using a second dataset where the 7 longest-branching sequences were removed. A monophyletic Euglenozoa was once again grafted to the positions where the euglenid, diplonemid, and kinetoplastid lineages were placed in ML trees inferred from this dataset, and in this case AU tests fail to reject euglenozoan EFL monophyly in any position (Table 3). Overall, the phylogeny of EFL provides strong evidence for differential loss of EFL and EF-1α in the kinetoplastid lineage, and the general failure of AU tests to reject euglenozoan EFL monophyly leaves open the possibility that differential loss after a single introduction of EFL may explain the entire distribution of EFL and EF-1α in Euglenozoa as a whole.

**Table 3:** Approximately Unbiased (AU) test p-values.

Topology, position of Euglenozoa	Dataset	
	EFL full	EFL short
LG, polyphyletic	0.454	0.444
LG, on kinetoplastids branch	0.001	0.164
LG, on *P. cantuscygni* branch	0.005	0.163
LG, on diplonemids branch	0.090	0.164
RtREV, polyphyletic	0.704	0.776
RtREV, on kinetoplastids branch	0.002	0.170
RtREV, on *P. cantuscygni* branch	0.000	0.170
RtREV, on diplonemids branch	0.039	0.167

## Discussion

Here we report the presence of EFL in the Euglenozoa, which occurs in a complex distribution that is not consistent with the known phylogenetic relationships of the organisms. Neither of these findings is unique to the Euglenozoa [4], [8], [9]; however, we also show that at least part of this complexity is best explained by differential loss of EFL and EF-1α from an ancestral state of co-occurrence rather than from multiple lateral transfer events. Three lines of evidence collectively support this interpretation. First, the monophyly of kinetoplastid EF-1α implies that this protein is ancestral in the kinetoplastids. Second, EFL sequences from *N. saliens* and *T. borreli* are closely related, implying that EFL was also present in their common ancestor. Third, analyses of other data consistently show that *T. borreli* and *N. saliens* are not sister taxa; rather, they belong to separate, consistently well-supported clades that have been named Parabodonida and Neobodonida, respectively [16], [20], [51], [52], [55]. Therefore *N. saliens* is more closely related to other neobodonids such as *R. nasuta* and *D. trypaniformis*, which, as we have demonstrated here, encode EF-1α. Although the branching order of kinetoplastid clades is somewhat variable, with notable differences in topology between SSU rRNA and heat shock protein phylogenies, neobodonids and parabodonids are always monophyletic groups, and are never sister to one another. The better-supported protein phylogenies favor a topology in which neo- and parabodonids branch as the deepest and next-deepest branches of the Metakinetoplastina (i.e. all kinetoplastids except the clade to which *P. amoebae* belongs), and their common ancestor is therefore also the ancestor of eubodonids and trypanosomatids (Fig. 3). Taken together, these lines of evidence suggest that there was a period of co-occurrence of EFL and EF-1α in the stem lineage of modern kinetoplastids, and the complex distribution of these proteins is due to differential loss or continued co-existence, which we cannot rule out until complete genome sequences of these organisms are available. To explain this distribution through lateral gene transfer, one would need to invoke two independent transfers, coincidentally from the same unidentified source, or a transfer to either *N. saliens* or *T. borreli* followed by a transfer between the two, neither of which seems especially likely. Given the alternatives outlined above, we consider the scenario of co-occurrence followed by differential loss to be the most parsimonious.

If differential loss after a period of co-occurrence can explain the complex distribution of EFL and EF-1α within the Metakinetoplastina, how well can it explain the complex distribution in the Euglenozoa as a whole? Here, there is no strong evidence for either lateral gene transfer or differential loss. The distribution and phylogeny of EF-1α indicate that this protein is ancestral in the Euglenozoa, and the distribution of EFL in deep-branching members of all three euglenozoan lineages suggests that this protein may also be ancestral. The phylogeny of EFL, however, is too poorly supported to make strong conclusions in either direction. Taken at face value, three separate clades of euglenozoan EFL imply three independent acquisitions, but without a clear identification of donor lineages for any of these putative transfers, this does not constitute evidence for lateral gene transfer. Furthermore, the separation of these lineages is weak, and several of the EFL topologies with a monophyletic Euglenozoa cannot be rejected. Given the evidence for differential loss in the kinetoplastids and the occurrence of EFL in all three euglenozoan lineages, we surmise that EFL's complex distribution in the Euglenozoa as a whole may be due entirely to differential loss.

Where did the euglenozoan EFL ultimately originate? The closest relatives of Euglenozoa are the Heterolobosea and Jakobida, with Heterolobosea being the most likely sister group [11], [56]–[58]. Only EF-1α sequences have been found in heterolobosean and jakobid taxa to date, including analyses of several EST projects described here, so at present there is no direct evidence for EFL in any excavate prior to the ancestor of Euglenozoa, although given the rapidity with which EFL has been discovered in diverse eukaryotes it would not be surprising if more excavate lineages are shown to possess it. Perhaps the anomalous EF-1α sequence of *A. rosea* is a hint that this species deserves further study. For both species in which EFL and EF-1α are currently known to co-occur, *T. pseudonana* and *B. ranarum*, EF-1α forms an unusually long branch (Fig. 1), similar to the EF-1α sequence of *A. rosea* (not shown).

The Euglenozoa are very isolated in the tree of eukaryotes from other lineages currently known to encode EFL, and therefore EFL's origin in the Euglenozoa is more simply explained by lateral gene transfer, but the demonstration here that differential loss plays a role in EFL's distribution needs to be considered more carefully at all levels of the tree. There is evidence that this might have played a part in the distribution of EFL in green algae, where there is support for the retention of the ancestral EF-1α but no support for a common origin of EFL genes in distantly related lineages [4]. Conversely, an analysis of EFL in diatoms has suggested a direct role for lateral transfer in that lineage [10]. The biggest question remains how lateral transfer and/or differential loss might have contributed to the distribution throughout eukaryotes as a whole. Without a robustly resolved phylogeny of EFL, which seems unlikely to emerge, we must remain open to the possibility that EFL's complex distribution is attributable to rampant lateral gene transfer; however, this study provides the strongest evidence to date that differential loss has also contributed to EFL's intriguing distribution.

Despite EFL's considerable sequence divergence from EF-1α (typically 40–45% sequence identity), it is considered likely to perform the same canonical function as EF-1α, namely cleaving GTP to deposit aminoacyl-tRNAs in the A site of the ribosome. This inference is based on two main observations. First, EF-1α's binding sites for aa-tRNAs, GTP, and its nucleotide exchange factor EF-1β are conserved in EFL: evolutionary rate shifts and divergence without rate shifts are confined primarily to non-binding sites. Second, EF-1α's function is essential, and as the protein with the closest similarity to EF-1α in EF-1α-lacking genomes, EFL is the most likely candidate for executing this function [3]. This leads to the question, why would one protein or the other be preferentially retained in different lineages? As yet there is very little data to address this question, but part of the answer may lie among the many additional cellular processes in which EF-1α has been implicated, such as actin bundling [59] and ubiquitin-dependent protein degradation [60], for which EFL might not share EF-1α's binding sites. Minor functional differences may also help to explain our conclusion that these two proteins are better able to co-exist than their present distribution suggests. For the majority of duplicate gene pairs, from which we can draw a loose analogy to EFL and EF-1α, one copy tends to be lost quite rapidly unless it undergoes sub- or neofunctionalization [61]. Much work is needed to determine whether functional differences exist, and if so, whether there may be adaptive significance to the complex distribution of EFL and EF-1α.
